# Artificial intelligence-driven gastrointestinal functional assessment: multimodal imaging, digital biomarkers, and real-time monitoring

**DOI:** 10.3389/fphys.2026.1778235

**Published:** 2026-03-25

**Authors:** Liucheng Li, Fang Lv, Chen Du, Lianjun Yang, Chengzhou Pa, Yunrui Dai

**Affiliations:** 1 Dermatology, YunNan Provincial Hospital of Traditional Chinese Medicine, Kunming, China; 2 Department of Critical Care Medicine, The First People’s Hospital of Kunming, The Affiliated Calmette Hospital of Kunming Medical University, Kunming, China; 3 Department of Hepatobiliary Pancreatic and Vascular Surgery, The First People’s Hospital of Kunming, The Affiliated Calmette Hospital of Kunming Medical University, Kunming, China; 4 Department of Magnetic Resonance Imaging, First People’s Hospital of Yunnan Province/Affiliated Hospital of Kunming University of Science and Technology, Kunming, China

**Keywords:** artificial intelligence, digital biomarkers, functional disorders, gastrointestinal physiology, multimodal imaging, real-time monitoring

## Abstract

Gastrointestinal (GI) functional disorders and chronic inflammatory diseases impose a substantial health burden, yet their assessment remains challenging because symptoms reflect dynamic interactions among motility, visceral sensation, immune–microbiome regulation, and brain–gut signaling. Artificial intelligence (AI) is rapidly reshaping GI functional medicine by enabling scalable, quantitative interpretation of complex data generated from multimodal imaging, physiological sensing, and real-world patient monitoring. This review synthesizes advances across three tightly connected pillars that map onto a physiology-informed “assessment-to-action” loop: (i) AI-assisted multimodal GI imaging for quantitative phenotyping and integrated diagnosis; (ii) AI-enabled discovery and validation of digital biomarkers that capture dynamic GI function in naturalistic settings; and (iii) real-time monitoring platforms that support early warning, longitudinal assessment, and adaptive management. We summarize representative applications in functional GI disorders, inflammatory bowel disease (IBD), and GI oncology, highlighting methodological themes including multimodal fusion, temporal modeling, uncertainty estimation, and explainable AI. We then discuss barriers to translation—standardization and interoperability, external validation under dataset shift, privacy and governance, and workflow integration—and outline practical directions for building clinically trustworthy AI systems for GI functional assessment. Collectively, physiology-centered AI approaches have the potential to transform GI care from episodic testing to longitudinal, mechanism-aware monitoring and personalized intervention.

## 1 Introduction

Gastrointestinal (GI) functional disorders and chronic GI diseases are difficult to diagnose and manage because clinical presentations arise from interacting disturbances in motility, visceral sensation, immune signaling, epithelial barrier function, and microbiome–host dynamics (Fuchs et al., 2022; Fuchs et al., 2023; Suri et al., 2024). In addition to physical symptoms, patients often experience psychological distress and impaired quality of life, highlighting the need for multidimensional assessment (Tanaka et al., 2023; Qiao et al., 2025).

Conventional tools—endoscopy, manometry, radiologic imaging, and histopathology—remain foundational but can be limited for functional medicine. Many approaches provide episodic “snapshots” rather than continuous physiological trajectories, and several are invasive, resource-intensive, or operator-dependent. In upper GI endoscopy, subtle precancerous or early malignant lesions can be missed; Artificial intelligence (AI) assistance has therefore been introduced to improve detection and reduce interobserver variability (Yu et al., 2021). Traditional biomarkers and scoring systems may also lack sensitivity for early diagnosis or for monitoring fluctuating disease states.

AI provides an integrative analytical layer that can connect heterogeneous measurements into a physiology-informed clinical loop. In gastroenterology, AI has shown promise from endoscopic image interpretation to multimodal integration of clinical and molecular data for diagnosis and prognosis (Wu et al., 2021; Labaki et al., 2024). The convolutional neural network (CNN) -based systems can achieve strong performance in lesion detection and characterization, enabling earlier intervention and individualized planning (Cuevas-Rodriguez et al., 2023; Spada et al., 2024; Quek and Ho, 2025). Beyond imaging, AI can help derive digital biomarkers from multimodal streams—including imaging, multi-omics, and patient-generated data—to reflect disease state and trajectory more precisely than episodic measures (Bragina et al., 2022; Wang et al., 2023).

This review is structured around three interrelated pillars aligned with gastrointestinal physiology and clinical decision-making: AI-assisted multimodal imaging for quantitative phenotyping, digital biomarkers as longitudinal indicators of functional status, and real-time monitoring systems for adaptive patient management. We further examine translational challenges and outline priorities for building clinically trustworthy AI systems. To enhance interpretability of the evidence base, studies are discussed according to their level of validation, including development-only models, internally validated studies, externally validated cohorts, and prospective or clinical impact evaluations.

The review was conducted as a structured narrative synthesis based on PubMed literature searches. Studies were selected for their relevance to gastrointestinal functional assessment, methodological transparency, and reported validation strategy. Multicenter and externally validated evidence was prioritized when available, while development-stage investigations were included when they offered clear physiological relevance and translational potential. To improve clinical interpretability, we organize the evidence by validation depth and translational maturity, distinguishing development-only retrospective studies, internally validated studies, externally validated multicenter studies, and prospective impact evaluations, including randomized studies when available. We also annotate the reported stage of clinical adoption (research-facing, pilot deployment, or routine workflow use), any regulatory clearance, and-when reported-economic or cost considerations. This structure is intended to avoid “catalog-style” listing and to clarify what is clinically actionable today versus what remains “on the horizon”. Key terminology is defined operationally in **Supplementary Table 1** to ensure consistency throughout the manuscript.

**Figure 1:**
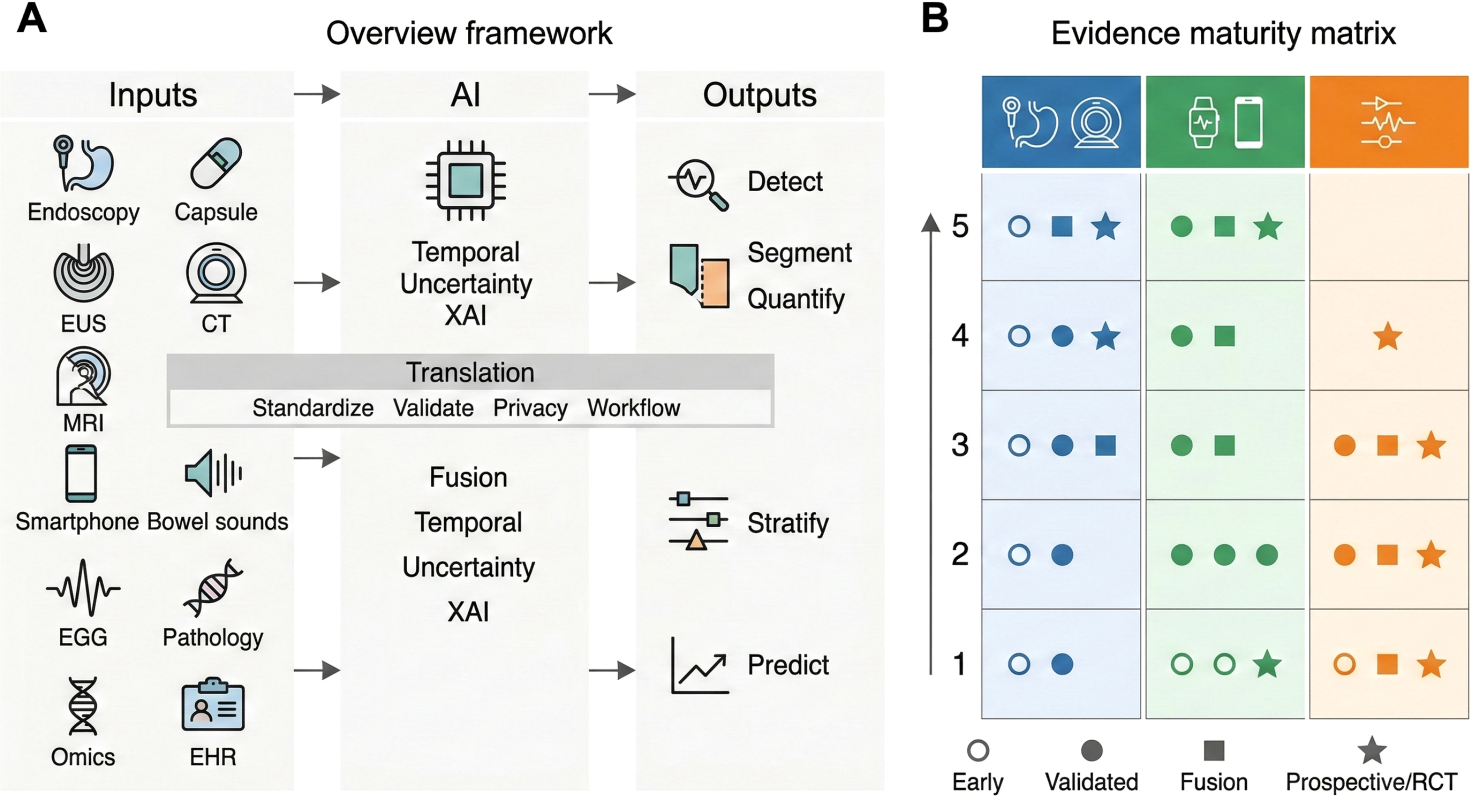
Integrated overview and evidence maturity map for AI-driven gastrointestinal functional assessment. **(A)** summarizes a physiology-informed pathway in which multimodal inputs-GI imaging (endoscopy/capsule/EUS, CT/MRI), patient-generated and wearable data, physiological signals, and clinical/molecular information—are integrated by an AI layer emphasizing multimodal fusion, temporal modeling, uncertainty awareness, and explainable AI. The resulting outputs support clinically relevant functions including detection/segmentation, quantitative phenotyping, risk stratification, and prediction, enabling longitudinal assessment and remote-care use cases. A translation checkpoint highlights practical requirements for adoption, including standardization, external validation under dataset shift, privacy governance, and workflow integration. **(B)** places representative approach classes across evidence tiers to compare validation depth across the three pillars (imaging AI, digital biomarkers, real-time monitoring); token density reflects representative maturity patterns rather than publication counts, helping distinguish clinically nearer applications from horizon concepts.

To orient the reader, **Figure 1** provides an at-a-glance synthesis of the review’s physiology-informed assessment pipeline and an evidence-tier maturity map across imaging AI, digital biomarkers, and real-time monitoring. Guided by this framework, we first focus on AI-driven multimodal imaging for quantitative GI phenotyping.

## 2 AI-driven multimodal imaging for quantitative GI phenotyping

### 2.1 Imaging modalities and physiological information content

Multimodal imaging is essential because GI diseases manifest across mucosal, mural, and extraluminal compartments and involve both structural and functional alterations. Conventional endoscopy provides direct mucosal visualization and enables biopsy and therapy, but it is invasive, often requires sedation, and may miss submucosal or extraluminal pathology. Endoscopic adverse events include cardiopulmonary complications, bleeding, perforation, pancreatitis, cholangitis, and infection, which limit its utility (Deb et al., 2022).

Less invasive capsule-based imaging is expanding mucosal assessment. For instance, Zhou et al. (2024) developed an oral near-infrared fluorescence capsule endoscope (NIFCE) that can excite and capture near-infrared (NIR) fluorescence images to identify subtle mucosal micro-injuries and submucosal lesions. In parallel, it acquires conventional white-light images to detect lesions with prominent morphological changes. They also constructed a collaborative system that enables multi-posture control of the NIFCE and provides long-term power supply, addressing the high power consumption associated with NIR emission. To further improve detection performance, a multimodal deep learning model, Multimodal-Retinex-Attention-YOLO (MRAY), was proposed to integrate white-light and fluorescence data for detecting subtle mucosal micro-injuries and submucosal abnormalities (Wang et al., 2025). Capsule platforms combining optical coherence tomography, miniature cameras, and therapeutic modules illustrate an emerging direction toward compact “diagnose-and-intervene” workflows for early lesion detection and guidance (Park et al., 2024).

Endoscopic ultrasound (EUS) complements luminal endoscopy by visualizing GI wall layers and adjacent structures and enabling tissue acquisition via fine-needle aspiration/biopsy (Tacelli et al., 2025). Its limitations include operator dependence, interobserver variability, and constraints related to penetration depth and patient tolerance. Cross-sectional imaging extends phenotyping beyond the lumen. Contrast-enhanced CT remains critical for tumor detection, staging, and vascular complications (Maggialetti et al., 2022; Sun et al., 2025). CT radiomics features extracted from the liver, peritumoral region, and arterial/venous phases have shown potential for distinguishing liver metastases, gastrointestinal cancers, and non-gastrointestinal primary tumors (Hou et al., 2025). In addition, contrast-enhanced CT–based radiological analysis has been used to predict lymphovascular invasion in gastric cancer and clinical outcomes (Fan et al., 2022; Li et al., 2022). MRI provides high soft-tissue contrast without ionizing radiation and supports functional characterization; advanced MRI techniques can detect early therapeutic response and characterize intestinal wall changes in Crohn’s disease and systemic sclerosis-related bowel involvement (Delaney et al., 2021; Bidani et al., 2022; Revheim et al., 2022; Paparo et al., 2024). Hybrid combinations (e.g., PET/CT with MRI) and multimodal ultrasound strategies (including CEUS) can integrate metabolic and vascular information, enabling more comprehensive phenotyping (Shi et al., 2022; Lipan et al., 2024). Emerging contrast strategies and AI-driven interpretation may further extend imaging toward molecular-level characterization, offering deeper insights into disease mechanisms (Tiwari et al., 2025; Wu et al., 2025).

### 2.2 AI tasks that matter clinically: from detection to quantification

In GI practice, imaging AI is most impactful when it improves reproducibility and links outputs to decisions. Deep learning methods have demonstrated strong performance for segmentation and classification across GI-relevant modalities, supporting delineation of tumors, polyps, and inflammatory changes (Pouly et al., 2020; Ramalakshmi et al., 2024). In endoscopy, CADe/CADx systems can assist in real time by reducing miss rates and standardizing characterization, improving both detection and diagnostic accuracy. However, the maturity of evidence differs substantially across applications, ranging from exploratory retrospective studies to prospective randomized evaluations. In 2024, Yuan et al. (2024) reported the first multi-center, sequential, double-blind, randomized controlled trial for esophageal squamous cell carcinoma (ESCC) using a CADe system. The results showed that the single lesion missed diagnosis rate in the AI-first group was 1.7%, while in the conventional-first group it was 6.7% (risk ratio 0.25; 95% confidence interval: 0.06 - 1.08; p = 0.079), and the missed diagnosis rate per patient in the AI-first group was 1.9%, while in the conventional-first group it was 5.1% (risk ratio 0.37; 95% confidence interval: 0.08 - 1.71; p = 0.40). In contrast, the detection rate at the first examination in the AI-first group was 1.8%, while in the conventional-first group it was 1.3% (risk ratio 1.38; 95% confidence interval: 1.03 - 1.86; p = 0.03). Although there was no significant difference in the single lesion and patient missed diagnosis rates for superficial ESCC and precancerous lesions, the CADe system significantly improved the detection rate. A meta-analysis showed that in gastric cancer detection, the CADe system has a sensitivity and specificity of up to 90%, supporting its use as a clinical expert assistance tool to compensate for the limitations of human experts (Kikuchi et al., 2024). Despite these encouraging results, most AI systems discussed in this review remain outside routine GI practice in many settings, and adoption depends on workflow integration and local governance.

Recent studies have demonstrated the potential of Vision Transformer (ViT) in classifying intestinal organoids. Among multiple deep learning architectures, ViT has shown superior performance in both accuracy and efficiency, particularly in real-time applications. This is particularly relevant for real-time monitoring and early detection of gastrointestinal cancers, such as colorectal cancer (CRC) (Cicceri et al., 2025). At present, these organoid-based approaches are predominantly research-facing, and their clinical translation will require external validation and prospective impact evaluation.

Architectural differences also influence performance. CNNs benefit from spatial inductive biases and may be more robust in gastrointestinal imaging tasks with limited datasets. ViTs model long-range dependencies through global attention but typically require larger-scale data for stable performance. Multimodal transformer frameworks further integrate imaging, clinical, and molecular inputs, enhancing cross-modal representation but remaining sensitive to dataset shift and modality imbalance. Standardized benchmarking and external validation are therefore essential for meaningful architectural comparison.

### 2.3 Robustness, uncertainty, and explainability in imaging AI

Real-world deployment is constrained by dataset shift: differences in devices, protocols, bowel preparation quality, and patient factors can degrade performance outside development settings. Performance variability may also arise from annotation bias, interobserver variability, and class imbalance, particularly in lesion detection tasks where rare pathological patterns are underrepresented. Such factors may inflate apparent performance in controlled settings while limiting real-world robustness. Accordingly, robustness should be interpreted alongside evidence tier, because development-only or single-center results often overestimate performance under real-world heterogeneity.

Methods for domain adaptation and explainable AI (XAI) aim to improve generalizability and transparency, addressing “black-box” concerns in medicolegally sensitive environments (Thirunavukarasu et al., 2023; Muhammad and Bendechache, 2024). For physiology-centered translation, model outputs should ideally provide calibrated uncertainty and interpretable evidence maps aligned with GI anatomy and pathophysiology. However, insufficient reporting of calibration metrics and uncertainty estimates remains common in current studies. From a clinical perspective, uncertainty-aware outputs are most useful when linked to explicit workflow actions rather than being reported as standalone technical metrics.

Despite encouraging diagnostic performance, many GI imaging AI studies remain retrospective and single-center, increasing susceptibility to spectrum bias and limiting generalizability to heterogeneous clinical populations. External validation across independent institutions is still relatively limited. These limitations help explain why many promising systems remain outside routine practice despite strong retrospective performance.

To provide a verifiable overview of clinically relevant imaging and endoscopy AI, we summarize representative applications, study designs, comparators, validation strategies, and primary endpoints in **Table 1**, highlighting the evidence level from development-only studies to prospective randomized evaluations.

**Table 1:** Representative AI applications in GI imaging and multimodal phenotyping.

Clinical domain	Modality	AI task	Study design / cohort	Comparator	Model output	Primary endpoint / validation	Evidence level	Translation status	Cost note
Upper GI neoplasia (superficial ESCC & precancerous lesions) (Yuan et al., 2024)	Upper GI endoscopy (WLE + non-magnified NBI)	CADe (real-time detection assistance)	Multicenter tandem double-blind RCT (12 hospitals; per-protocol)	AI-first vs routine-first (tandem same-day; order reversed)	Real-time alerts/visual prompts	Primary: miss rate (per-lesion & per-patient); safety AEs	Prospective / multicenter RCT	Clinical trial evidence; routine uptake not established	Cost-benefit not yet assessed (stated as needing further evaluation)
Suspected small bowel bleeding (Spada et al., 2024)	Capsule endoscopy (NaviCam SB + ProScan AI)	AI-assisted reading (lesion detection triage)	Prospective multicenter trial (14 European centers; n=133 analyzed)	Standard reading vs AI-assisted reading (blinded second reading)	AI-selected abnormal frames + marked regions	Diagnostic yield (P1/P2) non-inferiority & superiority; reading time reduction	Prospective multicenter (non-inferiority framework)	Pilot / early clinical deployment (device/software dependent)	Qualitative; procurement/licensing varies (not formally analyzed)
Gastric cancer (localized) (Li et al., 2022)	CECT	Radiomics + DTL for pre-op LVI prediction; integrated GRISK model	Retrospective cohort (train/test split; n=1062)	Radiomics marker vs DTL marker; GRISK vs components	LVI probability / GRISK score	AUC (train/test); prognosis association (PFS/OS)	Retrospective + internal validation	Research-facing / risk modelling	Not reported
Gastric cancer (LAGC) early recurrence (Ding et al., 2025)	Clinical + abdominal CT (radiomics) + post-op H&E WSI (pathomics)	Interpretable multimodal fusion risk stratification (RSA)	Multicenter cohorts (6 centers; n=1580) + multiple validations; primary outcome recurrence within 24 months	Clinical-only vs radiomic-only vs pathomic-only vs RSA	RSA risk score; SHAP-based interpretability visualizations	AUC: train 0.903 / internal 0.902 / external 0.884–0.889; notes not yet clinician-facing platform	Multicenter retrospective + external validation; additional validation using prospective trial dataset (retrospective analysis)	Pilot / specialized centers; not yet fully automated clinician-facing tool	Not reported
Colorectal cancer occult peritoneal metastasis (Miao et al., 2025)	Pre-op CT (tumor + visceral fat at L3) + clinical	Multimodal DL + clinical for occult PM prediction	Internal + external test sets	Radiologists vs model; tumor-only vs tumor+fat vs +clinical	Malignancy/PM probability; Grad-CAM regions	AUC internal 0.941; external 0.911; external testing	Retrospective	Research-facing / potential decision support	Not reported
Colorectal cancer (stage II–IV) (Loeffler et al., 2025)	Histology (H&E WSI) + ctDNA (MRD)	DFS/recurrence risk stratification; DL+MRD combined	Train DACHS (n=1766), validate GALAXY (n=1404); DL high vs low risk (HR reported)	MRD alone vs DL score alone vs combined	DL risk score (high/low) + MRD status	DFS stratification; combined DL+MRD improves stratification; chemo benefit signal in subgroup	Retrospective training + external validation; validation cohort embedded in prospective observational arm	Research-facing; hypothesis-generating for follow-up/ACT decisions	Not reported
Gastric cancer screening/auxiliary diagnosis (Yuan et al., 2023)	Tongue images (± tongue coating microbiome)	ML/DL diagnostic tool for GC	Prospective multicenter cohort + independent external validation (10 centers + 7 centers)	Compared with combination of eight blood biomarkers	Diagnostic probability (GC vs NGC; early GC/precancerous)	AUC: internal 0.88–0.92; external 0.83–0.88	Prospective multicenter + independent external validation	Pilot / screening-adjacent; not routine standard of care	Qualitative only (“more convenient/economical” language; no formal CEA)

## 3 Multimodal fusion for integrated diagnosis and mechanism-aware stratification

Single-modality models can miss complementary information required for GI phenotyping. Multimodal fusion integrates endoscopy, radiology, pathology, clinical text, and physiological data to improve diagnostic performance and support personalized decision-making (Ai et al., 2024). Evidence maturity varies across fusion applications, and many reports remain at development or internal-validation stages despite promising results. For instance, Ding et al. (2025) developed a multimodal fusion Risk Stratification Assessment (RSA) model that integrates clinical, radiological and pathological data. The RSA model significantly outperformed the models based solely on clinical data, radiology alone, or pathology alone in predicting early recurrence after gastric cancer surgery. The area under the curve (AUC) value in the training cohort was 0.903, while the internal validation was 0.902, and the external validation ranged from 0.884 to 0.889. The integrated multimodal RSA model effectively predicts the recurrence risk and prognosis of LAGC and enables precise stratification of patients and individualized postoperative management. Feature-level (intermediate) fusion often balances information richness with alignment stability and may outperform naïve early or late fusion strategies in complex classification tasks (Teoh et al., 2024).

Clinical studies support the value of fusion when aligned to actionable decisions. In Crohn’s disease, multimodal models integrating colonoscopy images, pathology slides, and clinical text improved classification and stratification compared with unimodal approaches (Jia et al., 2024). Fusion of endoscopic imaging with histopathology and clinical records can enhance detection and characterization of subtle mucosal changes and functional abnormalities (Alagappan et al., 2018). In hepatocellular carcinoma, a comprehensive analysis of transcriptomic, proteomic and metabolomic data identified six key genes (LCAT, PEMT, ACSL1, GPD1, ACSL4 and LPCAT1), which showed significant changes at both mRNA and protein levels and were highly correlated with lipid-related metabolites in HCC. This implies that identifying lipid-related metabolites as potential diagnostic biomarkers holds great promise for early detection and improving the clinical management of HCC (Dai et al., 2025). In GI oncology, multimodal fusion integrating endoscopic, pathological, and multi-omics information has improved early detection and individualized treatment decision-making (Ai et al., 2024; Miao et al., 2025). Importantly, fusion should prioritize clinically interpretable phenotypes and decision points; otherwise, complexity can outpace utility and hinder adoption. Practical deployment is further shaped by modality availability, interoperability, and governance requirements for multi-source clinical data.

Although internal validation is commonly reported, relatively few multimodal models have undergone rigorous external validation across independent institutions. This limits confidence in their robustness under dataset shift and heterogeneous clinical workflows. Prospective multicenter evaluation remains necessary before multimodal fusion systems can be considered clinically reliable. As a result, many fusion models remain translational rather than routinely implemented despite encouraging retrospective performance.

## 4 Digital biomarkers in GI functional medicine

### 4.1 Definition and GI-focused taxonomy

Digital biomarkers are quantifiable indicators of physiological or behavioral states derived from digitally captured data (e.g., wearables, smartphones, sensor platforms, connected diagnostics). They enable continuous, real-time, and often non-invasive monitoring in naturalistic settings (Motahari-Nezhad et al., 2022). Their adoption accelerated during the COVID-19 era, highlighting feasibility and value for remote assessment (Crawford and Serhal, 2020). Validation depth and clinical uptake vary across GI digital biomarker approaches.

A GI-focused taxonomy facilitates endpoint design. Behavioral biomarkers include symptom diaries, dietary logs, sleep, and activity patterns (Ryu et al., 2021; Motahari-Nezhad et al., 2022). Physiological biomarkers encompass organ-relevant biosignals and functional measures captured continuously—potentially including bowel sound features and electrogastrographic measures—providing objective correlates of motility and autonomic regulation (Soangra et al., 2023; Takkavatakarn and Hofer, 2023). Molecular or multi-omics-associated digital biomarkers integrate laboratory/omics measurements with digital platforms for refined phenotyping and prognostication (Ni et al., 2025; Wotman et al., 2025). Multimodal panels combining behavioral and physiological streams may provide robust endpoints for disease monitoring and individualized care (Lucarelli et al., 2023; Epelde, 2025), which is particularly relevant for GI disorders where symptoms fluctuate and real-world functioning is clinically meaningful (Botros et al., 2022). Implementation also depends on data quality, privacy governance, and practical resource requirements across platforms.

Because digital biomarkers are clinically meaningful only when validated and linked to actionable clinical decisions, **Table 2** summarizes representative GI digital biomarker and real-time monitoring studies, reporting the sensing modality, cohort and study design, comparator, validation strategy, and primary endpoints. To improve interpretability, we summarize evidence tier and reported implementation status where such information is available.

**Table 2:** Representative AI-based digital biomarkers and real-time monitoring in GI functional medicine.

Sensing modality	Clinical context	Study design / cohort	Comparator	Digital output	Validation / primary endpoint	Evidence level	Translation status	Cost note
Acoustic bowel sounds (AI-assisted auscultation system) (Liu et al., 2024)	Acute pancreatitis: early enteral nutrition-associated diarrhea (ENAD) risk prediction	Single-center prospective observational study; n=133; internal validation via Bootstrap; model uses random forest	Not framed as device-vs-standard RCT; model-based risk stratification for ENAD	ENAD risk score/probability from real-time bowel sound features	AUC 0.904 (95% CI 0.817–0.997); accuracy/sensitivity/specificity reported	Prospective observational + internal validation	Pilot / research-facing (single-center; no external validation)	Not reported
Acoustic bowel sounds (wearables/smartphone acquisition; AI analytics) (Sood et al., 2025)	IBD diagnosis/characterization/flare monitoring (reviewed evidence)	Narrative review; summarizes studies with 16–100 participants; reports performance ranges and limitations	Across-study comparisons (IBD vs healthy/IBS; model families)	Acoustic features/indices; model outputs for activity/flare/differential diagnosis (review summary)	Reported accuracies 88–96%, AUC ≥0.83; generalizability limited; no prospective validation	Review (evidence heterogeneous; mostly small cohorts)	Research-facing / early-stage	Qualitative only (no formal economic evaluation)
Location-aware ingestible microdevices (wireless tracking/localization) (Sharma et al., 2023)	Quantitative GI dynamics monitoring (transit-time evaluation; motility disorders; targeting interventions)	*In vivo* large-animal demonstration; real-time 3D tracking with mm-scale resolution	Not a clinical comparator trial; positions relative to limitations of endoscopy/manometry/CT/scintigraphy described	Real-time device location/time series; GI transit metrics; potential for targeted sensing/therapy	Demonstrates feasibility of real-time tracking/localization; translational value framed for ambulatory/at-home concepts	Preclinical/technical validation (animal model)	Horizon / preclinical-to-early translation	Not reported
Implantable GI bioelectrical activity acquisition (64-channel NFC system) (Javan-Khoshkholgh and Farajidavar, 2019)	Gastric slow-wave (HR mapping) for dysrhythmias (e.g., gastroparesis/functional dyspepsia)	Engineering/benchtop validation; implantable unit + wearable unit + stationary unit; wireless power/data telemetry	Not a clinical outcome comparator	Multichannel gastric electrical activity signals; real-time monitoring via GUI	Demonstrates successful data communication (≤5 cm) and verified signal transmission *in vitro*	Technical/benchtop validation	Research-facing / early-stage	Not reported
Ingestible electronic devices (telemetric capsules with sensors ± interventional functions) (Thwaites et al., 2024)	Ingestible electronic devices (telemetric capsules with sensors ± interventional functions)	Ingestible electronic devices (telemetric capsules with sensors ± interventional functions)	Ingestible electronic devices (telemetric capsules with sensors ± interventional functions)	Ingestible electronic devices (telemetric capsules with sensors ± interventional functions)	Ingestible electronic devices (telemetric capsules with sensors ± interventional functions)	Ingestible electronic devices (telemetric capsules with sensors ± interventional functions)	Ingestible electronic devices (telemetric capsules with sensors ± interventional functions)	Ingestible electronic devices (telemetric capsules with sensors ± interventional functions)
Wireless biodegradable implantable electronics (review) (Cho et al., 2025)	Real-time clinical monitoring with transient implants (incl. GI applications such as leak detection; broad scope)	Review of materials + wireless comms/power + applications	—	Wireless telemetry/power approaches; example application classes (incl. digestive system)	Conceptual/technology readiness discussion; not a single clinical validation study	Review	Horizon / enabling technology	Not reported

### 4.2 Discovery and validation pipeline: what makes a “clinically usable” biomarker

AI supports discovery by mining high-dimensional, multimodal datasets. Across biomedical research, AI has integrated multi-omics, imaging, and histopathology to identify predictive and prognostic signatures (AlOsaimi et al., 2025; Hussen et al., 2025; Singh et al., 2025). In GI contexts, AI has been applied to microbiome profiles to identify microbial biomarkers linked to intestinal diseases (Lin et al., 2021) and to metabolomic profiles for differentiating disease subtypes and functional states (Yu et al., 2025). Non-invasive physiological sources are under active development; bowel sound analysis has been proposed for monitoring IBD-related activity (Sood et al., 2025). Digital pathology also contributes to tumor classification and prognosis prediction in GI oncology (Kuntz et al., 2021; Yoshida and Kiyuna, 2021). Validation depth is heterogeneous across these modalities, and reported performance should be interpreted in light of study design and cohort diversity.

AI-powered digital biomarkers are evolving rapidly, especially in the context of GI functional disorders. ViT and other deep learning models are revolutionizing how we classify and monitor intestinal organoid phenotypes, particularly for studying diseases like CRC. ViT’s superior performance in organoid image classification supports its application in early cancer detection and therapeutic monitoring (Cicceri et al., 2025).

Rather than focusing solely on biomarker discovery, the narrative should emphasize the full validation pathway required for clinical use, including standardized data acquisition and labeling, modeling strategies with appropriate feature selection under collinearity and low signal-to-noise conditions (Vashistha et al., 2023; Pateras et al., 2024), internal validation with calibration assessment, and external validation in independent cohorts to establish generalizability (Kuntz et al., 2021). Translation into practice further requires evidence of analytic validity, clinical validity, and clinical utility, ideally demonstrated through prospective multicenter studies and evaluation frameworks aligned with regulatory expectations (Diamantopoulos et al., 2025). Emerging work using tongue image–based AI modeling for gastric cancer exemplifies how non-invasive digital phenotypes can complement conventional biomarkers within this validation paradigm (Yuan et al., 2023). Persistent challenges include dataset shift across populations, lack of standardized endpoints, and the need for explainable outputs to support clinician trust and adoption (Dockès et al., 2021; Ng et al., 2023). Where available, reporting on implementation considerations can further inform translational appraisal.

Despite increasing methodological sophistication, many digital biomarker studies remain limited by small sample sizes and short follow-up periods. Such constraints raise concerns regarding statistical power, reproducibility, and the risk of overfitting in high-dimensional feature spaces. These limitations underscore the need for larger, longitudinal, and externally validated cohorts to establish robust and clinically actionable digital endpoints.

### 4.3 Clinical use cases: early warning and therapy monitoring

Digital biomarkers enable early warning and longitudinal management by capturing subtle deviations that may precede clinical events. In IBD, multiscale modeling and “digital twin” concepts have been explored to predict treatment response and disease trajectory by integrating molecular and clinical information, including inflammatory mediators such as IL-6 (Pinton, 2022; Pinton, 2023). The level of clinical validation and implementation experience, however, varies across these approaches. Digital biomarkers can also quantify GI physiology more directly; implantable systems for real-time gastric electrical activity monitoring demonstrate feasibility of continuous sensing with wireless telemetry (Javan-Khoshkholgh and Farajidavar, 2019). In clinical trials, remote monitoring and real-world evidence are increasingly incorporated to complement traditional endpoints and improve relevance to patient care (Sedano et al., 2025). In GI oncology, image-derived digital pathology biomarkers can inform treatment stratification and predict therapeutic efficacy (Liu et al., 2024). To scale these applications, standardized endpoint definitions, interpretable outputs, and integrated workflows are required, together with safeguards for privacy and fairness (Zhao et al., 2025). In practice, translation is also shaped by follow-up burden, data governance, and resource considerations. Prospective evaluations that link early-warning signals to actionable care pathways will be particularly informative for assessing clinical utility.

## 5 Real-time monitoring and AI analytics platforms

### 5.1 Devices and sensing ecosystems

Real-time monitoring extends GI assessment beyond the clinic by enabling continuous capture of physiological streams and patient-generated data. Wearable sensors can capture biomechanical and autonomic signals that may correlate with GI motility and recovery (Du et al., 2024). Wireless multimodal platforms integrating acoustics and biopotentials illustrate multi-signal capture potential (Moon and Lee, 2023), while practical deployment is shaped by communication bandwidth and power constraints; hybrid energy-harvesting strategies have been proposed to support sustained IoT sensing (Lee et al., 2024). Feasibility and validation depth differ across device classes, and reported performance should be interpreted with attention to study context and deployment setting. Portable sensing approaches, including plasmonic sensors, enable sensitive detection of disease-related molecules with potential GI relevance (Yılmaz et al., 2025), and wearable biochemical sensing linked to IoT platforms extends monitoring to biomolecular parameters (Shahzad et al., 2024). Ingestible devices enable minimally invasive internal sensing but require solutions for localization and clinical integration (Thwaites et al., 2024). Wearable ultrasound supports noninvasive deep tissue monitoring (Fang et al., 2025), and biodegradable implantable electronics offer transient monitoring approaches that reduce infection risk and avoid retrieval (Cho et al., 2025). Practical implementation is additionally influenced by usability, maintenance requirements, and operational constraints in real-world care pathways. Resource implications vary substantially across platforms and are not consistently reported in the current literature.

### 5.2 Streaming analytics, anomaly detection, and interpretability

Continuous monitoring produces real-time data streams requiring robust preprocessing, calibration, and temporal modeling. Approaches include noise reduction, normalization, dimensionality reduction, and handling missingness/sensor faults (Beck et al., 2024; Wang et al., 2025; Yang et al., 2025). Multimodal fusion can improve representation learning for detection and prediction tasks (Bhak et al., 2025; Sang et al., 2025). For early warning, anomaly detection methods may model normal physiological patterns and flag deviations. Autoencoder-based architectures can detect anomalies via reconstruction error (Oluwasanmi et al., 2021); Generative Adversarial Networks (GAN) -based approaches can learn normal distributions for efficient anomaly recognition (Song et al., 2025); and ensemble learning can improve robustness in multifactorial classification (Bellamoli et al., 2023). Graph-based approaches may capture relational structure among multimodal features (Lecca and Lecca, 2023). Clinically, interpretability is essential: XAI methods (e.g., SHAP-like explanations) support clinician review of alerts and predictions (Zhou et al., 2024; Alzakari et al., 2025). Edge computing can reduce latency and limit raw-data transmission (Apiecionek, 2024), while privacy-preserving collaboration (e.g., federated learning) supports cross-institution refinement without exposing raw sensitive data (Karthick Raghunath et al., 2025). In practice, the value of early-warning analytics depends on alert calibration and how outputs map onto specific clinical actions and escalation pathways.

The integration of ViT models into real-time monitoring platforms allows for efficient and accurate detection of gastrointestinal anomalies. The real-time monitoring of intestinal organoids using ViT can significantly enhance early detection of diseases, paving the way for personalized interventions (Cicceri et al., 2025). At present, many such demonstrations are reported in limited settings, and broader clinical translation will benefit from external validation and prospective evaluation. Workflow integration and operational requirements remain important determinants of real-world adoption.

### 5.3 Clinical workflow integration and remote care

Monitoring becomes transformative when it changes decisions and improves outcomes. AI-assisted bowel sound analysis has been used to predict enteral nutrition-associated diarrhea risk in acute pancreatitis, enabling timely intervention and individualized management (Liu et al., 2024). Wearable and ingestible devices may support longitudinal management by tracking functional parameters and enabling earlier response to deterioration (Yang et al., 2024). In GI oncology, AI-integrated multimodal imaging supports early detection and monitoring (Liu et al., 2025). Telemedicine and remote follow-up are natural deployment contexts for validated digital endpoints (Yim et al., 2024), and AI systems integrating clinical text with endoscopic imaging can support remote classification and follow-up within coherent workflows (Sharmila and Geetha, 2025). Physiological monitoring studies have explored gastric slow-wave irregularities as objective markers of altered gastric motility (Araki et al., 2021), and ingestible sensors capable of attaching to mucosa and recording motility for days illustrate a pathway toward continuous, smartphone-accessible internal monitoring (Sharma et al., 2023). Interoperability, governance, usability, and clinician training remain decisive for adoption, ensuring effective implementation of these technologies (Ning et al., 2024; Yim et al., 2024). In practice, the level of clinical evaluation and implementation experience varies across monitoring solutions. Clear responsibility pathways for alerts and follow-up actions can facilitate safe integration into routine care. Operational considerations, including resource requirements, may influence adoption in different clinical settings.

## 6 From performance to practice: translational readiness and implementation

### 6.1 Beyond AUC: toward clinical readiness

Although discrimination metrics such as AUC are widely reported, high performance alone does not establish clinical readiness. For AI systems in gastrointestinal functional assessment, evaluation must extend beyond accuracy to address calibration, clinical utility, robustness, and implementation feasibility. Where available, evidence tier and reported implementation status can help contextualize performance claims.

Reliable calibration and reproducibility across heterogeneous datasets are essential, particularly when outputs inform surveillance or treatment decisions. Clinical utility requires external validation and decision-analytic evidence of added benefit over standard care. Robustness under dataset shift - across institutions, devices, and patient populations - remains critical, while interpretability, uncertainty estimation, and workflow compatibility support responsible integration. Practical deployment also benefits from clearly defined decision points and escalation pathways linked to model outputs.

Currently, much of the evidence is based on development-stage or internally validated models, with limited prospective multicenter and outcome-linked studies, underscoring a persistent translational gap. These considerations may help explain why routine clinical adoption remains variable despite encouraging retrospective results.

We therefore propose a clinical readiness framework (**Supplementary Table 2**) to guide structured appraisal beyond traditional performance metrics and strengthen translational alignment.

### 6.2 Standardization, interoperability, and data quality

Protocol differences across centers can alter derived features and degrade external performance. Imaging variability can produce substantial radiomics feature deviations and reduced AUC in cross-institution validation, emphasizing the need for harmonized acquisition, calibration, and reporting standards (Feng et al., 2024). Interoperability across devices and electronic health records is equally important for scalable deployment. Clear acquisition and reporting practices also make results easier to compare across studies and centers. Routine monitoring of data quality can help reduce avoidable variability over time.

### 6.3 Privacy, ethics, and governance

Large-scale health data introduces privacy risks including inference and inversion attacks. Federated learning reduces raw-data sharing (Hanser, 2023), and differential privacy provides formal protection through controlled noise (Shen and Zhong, 2021). Ethical deployment requires informed consent, transparency, accountability, bias mitigation, and validation across diverse populations (Sieren et al., 2025). Governance must address secondary use and data sharing (Larson et al., 2020), particularly for highly identifying physiological signals (Kunin et al., 2020). In multi-center settings, consistent governance practices can facilitate collaboration and trust. More explicit reporting of governance details can also support clearer interpretation of real-world feasibility.

### 6.4 Translation barriers: generalizability, regulation, and human factors

Robustness across clinical environments remains challenging given heterogeneous devices, protocols, and patient populations (Zhang and Wang, 2022; Meystre et al., 2023; Hudson et al., 2024; Malakar et al., 2024; He et al., 2025). Regulatory pathways may lag behind innovation; lack of standardized evaluation frameworks for multimodal products contributes to a persistent “last mile” gap (Gao et al., 2025). Interpretability and training are central: many gastroenterologists report difficulty understanding AI decision bases in attention-driven models (Rimondi et al., 2024). Standardized operational protocols and clarified clinical responsibility are essential (Fass et al., 2024; Li, 2025). Strategic planning for gastroenterology services will shape adoption trajectories (De-Madaria et al., 2022). Privacy-preserving multicenter learning can support continuous refinement while protecting confidentiality (Wu et al., 2025). In practice, workflow fit and clarity of responsibility often influence adoption as much as model performance. Studies that report how systems are used in real settings can provide especially useful context.

### 6.5 Research priorities

Key priorities include externally validated, outcome-linked evaluation; uncertainty-aware and explainable decision support; multimodal fusion methods capable of handling modality imbalance and temporal dynamics (Sharmila and Geetha, 2025); and standardized digital biomarker definitions with reproducible validation protocols. Real-time monitoring is likely to evolve toward closed-loop systems combining sensing, prediction, and adaptive intervention, enabled by miniaturization and biocompatible materials (Guo et al., 2025; Luo et al., 2025). Alongside clinical endpoints, it may be helpful to report practical requirements such as staffing, infrastructure, and follow-up burden when available. More consistent study reporting would also make it easier to compare approaches across rapidly evolving toolsets.

## 7 Conclusion

AI-driven multimodal imaging, digital biomarkers, and real-time monitoring are converging to provide a quantitative, longitudinal approach for GI functional assessment. Achieving clinical impact requires standardized data, interoperability, external validation, privacy-preserving collaborations, and seamless integration into workflows. With co-development and implementation-focused designs, AI has the potential to enhance diagnosis, monitoring, and personalized management across GI disorders. However, routine clinical use remains variable across settings, and many approaches require further prospective, outcome-linked evaluation.

In summary, AI, particularly ViT, may improve early detection, diagnosis, and personalized treatment, shifting assessment from episodic testing to continuous, mechanism-informed monitoring and adaptive management. In the near term, the most readily deployable impact is likely to come from well-defined applications with robust validation and clear clinical endpoints, while several multimodal and continuous monitoring approaches remain “on the horizon.”

## Supplementary material

Supplementary Table 1 Glossary of key terms.

Supplementary Table 2 Clinical readiness evaluation framework for AI in GI functional assessment.
