# Supplementary material for: Artificial intelligence-driven gastrointestinal functional assessment: multimodal imaging, digital biomarkers, and real-time monitoring
Source: Front Physiol. 2026 Mar 25;17:1778235. doi: 10.3389/fphys.2026.1778235 (PMC13056675; doi:10.3389/fphys.2026.1778235)
Supplement: Supplementary Table 1 — Glossary of key terms. [file Table1.docx]

**Supplementary Table S1. Glossary of Key Terms**

| **Term** | **Operational Definition in This Review** |
| --- | --- |
| Artificial Intelligence (AI) | Computational systems designed to perform tasks requiring human-like pattern recognition or decision support. |
| Machine Learning (ML) | A subset of AI involving algorithms that learn patterns from data without explicit programming. |
| Deep Learning (DL) | A class of ML methods based on multi-layer neural networks for high-dimensional representation learning. |
| Multimodal Imaging | Integration of two or more imaging modalities (e.g., endoscopy, CT, MRI, ultrasound) for complementary structural or functional information. |
| Multimodal Fusion | Computational integration of heterogeneous data streams (imaging, clinical, molecular, physiological) into a unified predictive model. |
| Digital Biomarker | A quantifiable physiological or behavioral measure derived from digitally captured data to reflect health status or disease activity. |
| Functional Assessment | Quantitative evaluation of gastrointestinal physiological processes (e.g., motility, secretion, barrier function, neuro-immune interaction) rather than structural abnormalities alone. |
| Dataset Shift | Degradation in model performance due to differences between training and deployment data distributions. |
| Clinical Readiness | The extent to which an AI system demonstrates analytic validity, clinical validity, clinical utility, robustness, and implementation feasibility. |
| Clinical Utility | Evidence that model-guided decisions improve patient management or outcomes compared with standard care. |
| Prospective Impact Study | Evaluation of an AI system in real-time clinical use to measure changes in decision-making or patient outcomes. |
