# Supplementary material for: Artificial intelligence-driven gastrointestinal functional assessment: multimodal imaging, digital biomarkers, and real-time monitoring
Source: Front Physiol. 2026 Mar 25;17:1778235. doi: 10.3389/fphys.2026.1778235 (PMC13056675; doi:10.3389/fphys.2026.1778235)
Supplement: Supplementary Table 2 — Clinical readiness evaluation framework for AI in GI functional assessment. [file Table2.docx]

**Supplementary Table S2. Clinical Readiness Evaluation Framework for AI in GI Functional Assessment**

| **Dimension** | **Key Question** | **Example Metrics** |
| --- | --- | --- |
| Analytic validity | Is the model technically reliable? | AUC, calibration, Brier score |
| Clinical validity | Does it predict meaningful clinical states? | External validation, subgroup analysis |
| Clinical utility | Does it improve decisions? | Decision curve analysis, NRI |
| Robustness | Is it stable across centers/devices? | Cross-site testing |
| Interpretability | Can clinicians understand outputs? | SHAP maps, attention maps |
| Workflow integration | Can it fit into clinical pathways? | Time-to-decision, usability |
| Prospective impact | Does it change outcomes? | RCT, impact studies |
| Governance | Is privacy/fairness addressed? | Federated learning, bias audits |
